# Molding Binder Influence on the Porosity and Gas Permeability of Ceramic Casting Molds

**DOI:** 10.3390/ma13122735

**Published:** 2020-06-16

**Authors:** Pawel Wisniewski, Ryszard Sitek, Aleksandra Towarek, Emilia Choinska, Dorota Moszczynska, Jaroslaw Mizera

**Affiliations:** Faculty of Materials Science and Engineering, Warsaw University of Technology, Wołoska 141, 02-507 Warsaw, Poland; ryszard.sitek@pw.edu.pl (R.S.); aleksandra.towarek.dokt@pw.edu.pl (A.T.); emilia.choinska@pw.edu.pl (E.C.); dorota.moszczynska@pw.edu.pl (D.M.); jaroslaw.mizera@pw.edu.pl (J.M.)

**Keywords:** casting molds, precision casting, gas permeability, silica sand, µCT, binders, nanosilica dioxide

## Abstract

The investment casting process is widely used in the aerospace industry to produce complex engine parts. The article determines the properties of quartz powders, nanosilica dioxide binders, and multilayer samples of ceramic casting molds. The properties of spherical molds obtained using an alcohol-water system derived from hydrolyzed ethyl silicate (ZKE) and LUDOX PX-30 (type Q1) were compared with those obtained in water systems derived from Remasol Plus and Remasol Premium binders (type Q2). The spherical samples are composed of seven layers made with the use of an immersion-sprinkling method. To assess the properties of the molds, X-ray microscopy (µCT), scanning electron microscopy (SEM), and gas permeability analysis over a temperature range of 20–950 °C were utilized. The binder type is proven to affect the properties of the casting mold samples. The material obtained in the water system, Q2, has advantageous properties such as a high porosity and gas permeability.

## 1. Introduction

Precision casting has been widely developed by the arms industry, for the production of aircraft engine components. Components of modern aircraft engines manufactured using the lost-wax casting method were proved to be reliable and their production is now under a strictly controlled technological regime [1]. Ceramic molds with firmly specified properties, made from alloys such as CMSX4 and CMSX6, allow manufacturing precision castings with the Bridgman method. This method allows to obtain a specific microstructure in the mold, in the form of mono- or poly-crystalline castings with specific grain orientation [2,3]. This type of approach is used for manufacturing heavily stressed parts for aircraft engine sections, such as high-pressure turbines. The blades often operate at critical temperatures up to the melting point of the alloy. The lost-wax casting method enables creating cooling ducts, which are therefore essential. During the manufacturing process, the wax model contains a properly shaped ceramic core. Due to the use of ceramic molding materials and the technologically sophisticated alloys, it is possible to obtain precise molds with complex shapes and a low roughness (Ra = 1.0–5.0 µm) [4]. Ceramic molds, which are properly constructed and manufactured, allow a directional heat absorption from the volume of the crystallizing alloy. This allows to achieve a custom microstructure with the desired fraction of the particles in the desired orientation. Therefore it is possible to obtain equiangular column grains or whole elements consisting of a single crystal.

Production of the multilayer ceramic casting molds used for precision casting with the lost-wax casting method is a multi-step technological process. It begins with making two ceramic casting slurries, one for the face layer and latter for the construction layers. The molding slurry is a mixture of powders. [5]. Oxide powders with appropriate particle sizes make binders, usually water-based dispersions containing colloidal silicon oxide particles, organic additives (anti-foaming and wetting agent) and a particle size modifier [6]. The molding compound is formed in a reactor where the powders and binder are homogenized through continuous mixing.

Subsequently, molding compound is applied to a previously prepared wax model. The model is sprinkled with a dry molding powder after draining off the excess. Ceramic molds have a varying number of layers depending on the production requirements, such as alternating layers and sprinkling. The number of layers varies from five to eight, however, for monocrystalline castings, the number of layers can be as high as 12 [7]. The sealing layer is a finishing layer of the mold, preventing the powder topping. The ceramic casting molds are manufactured in an environment with controlled temperature and humidity, which allows the molds to dry easily without a risk of breakage and reduced amount of possible cracks [8].

Dry and finished ceramic molds are transferred to autoclaves, where the wax model is melted in a hot steam environment. Then the mold derived from the model is subjected to two heat treatment steps: burning and annealing. Once the annealing process is complete, the mold is filled with liquid metal under vacuum conditions in a casting furnace. The cast is removed from the ceramic shell after solidification.

The materials used for the production of ceramic casting molds determine their physical and mechanical properties. The molds are expected to be creep resistant, heatproof, heat resistant, have high mechanical strength, and high gas permeability. The mold’s first layer interacts directly with liquid metal and should be erosion resistant and non-reactive with the alloy. To refine the casting microstructure, a grain size modifier is included in the system, and its reaction with the alloy is expected to produce future crystallites [9].

The particle size of the powders and materials used in the binders has a direct influence on the porosity of the casting molds. The size, type, and distribution of pores can increase or decrease the mechanical strength of the mold. It also plays an important role in the thermal conductivity through the walls of the ceramic casting molds [10].

The scope of this study is to examine the materials used to obtain samples of molds and ceramics of casting molds in the raw state. Moreover, the gas permeability of the materials after thermal treatment study will be examined. Scanning electron microscopy (SEM), X-ray microtomography (µCT), and gas permeability measurements of the molds in a temperature range from 20 to 950 °C are performed to characterize the obtained molds. Scanning electron microscopy is an essential tool for imaging the morphology of the ceramic molds used in foundry and aerospace industries. µCT scanning allows to reconstruct the samples in the form of 3D images. Gas permeability is less used in investment casting, however it enabled characterization of the ceramic shell molds.

The general aim of the study is to compare the properties of two molds obtained by two different mechanisms: alcohol and water molding (Q1) and water molding (Q2). We address the possibility of replacing hydrolyzed ethyl silicate (ZKE) with commercial water binders such as Remasol Plus and Remasol Premium.

The ZKE binder contains alcohol, therefore it has a detrimental effect on the mucous membranes of its users and worsens the working conditions. What is more, it has a deleterious impact on the environment. On the contrary, colloidal silicas such as Remasol are non-toxic and non-flammable. Therefore, ZKE binder should be withdrawn from the modern foundry practice and replaced with more effective water systems providing better quality of shell molds [11,12,13,14,15,16,17,18].

## 2. Materials and Methods

Two types of multilayer, spherical ceramic molds were made with the use of an immersion method. Each sample consisted of seven layers; the first layer (layer 1), the sealing layer (layer 2), and five structural layers. The samples were made on a technical scale using the “immersion-sprinkling” method following the foundry’s technological mechanism. The samples obtained with this method were used for gas permeability measurements. Silica powder (Remet, UK) with a particle size of 325 Mesh (approximately 30 µm) was used to make the ceramic slurry. The polymer binders included: ZKE, LUDOX PX30, Remasol Plus, and Remasol Premium (Remet, UK) containing colloidal SiO_2_. Two particle sizes of silica sand were used for sprinkling: 0.1–0.3 mm for the first two layers, and 0.5–1.0 mm for the structural layers. The material composition of the moulding mixes and sprinkles is presented in Table 1.

Basic properties of the: silica meal, silica sand as a sprinkling material and polymeric binders were determined. The silica powder, the sand applied to the layers, and the mold samples were examined using an AXIO Scope.1 Zeiss (Oberkochen, Germany) light microscope and Hitachi (Tokyo, Japan) SU70 scanning electron microscope at an acceleration voltage of 4–5 kV.

Thermogravimetric analysis (TGA) of the silica sampled were performed in the air atmosphere with using Q5000 analyzer (TA Instruments, New Castle, DE, USA). Powder samples were scanned in the temperature range from 20 to 1000 °C with the heating rate 10 °C/min.

For the polymeric binders parameters such as: pH, solid phase composition (c), and discharge time from Zahn’s No 4 drawing cup as a standard method for viscosity evaluation used in precision casting in industrial conditions were determined. The binder acidity was monitored using a pH meter from Sension-1 Hach (Wien, Austria).

The gas permeability was determined using a homebuilt testing device designed and manufactured at the Faculty of Materials Science and Engineering of the Warsaw University of Technology. This was specifically manufactured to determine gas permeability in the industrial foundries.

The cut raw and sintered mold samples were prepared for tomographic tests. The samples were heated in a chamber furnace in the 950 °C/h. The heating ratio was 5 °C/min. After the process samples were cooled in the furnace. Mold samples were placed in Skyscan (Edinburgh, UK) 1172 X-ray microtomography. The samples were uniform in size and density, as this affects the extent to which they can be penetrated by the X-rays.

The optimum voltage of the lamp, the measuring step, and exposure time were set for the sample. These parameters are based on the degree of the X-ray radiation permeability of the examined samples. Scans were made in a rotation range from 0 to 180°. A total of 450 projections were made for each sample. The scanning parameters are given in Table 2.

The chemical composition was analysed using a Bruker (Karlsruhe, Germany) S4 Explorer X-ray fluorescence spectrometer, equipped with a Rh X-ray lamp with a copper anode; Cu, Pb, and Al filters; 0.23°, 0.46°, 1°, and 2° collimators; and crystals of LiF200, Ge, PET, and XS-55. Reconstruction and structure analyses were completed using Bruker software (NRecon version 1.7.3.0, DataViewer version 1.5.4.6, CTVox version 3.3.0 r1403, and CTAn version 1.17.7.2+). The difference of the X-ray absorption between the particles of solid phase particles and the hardened binder allowed to calculate the fraction of the solid phase (particle share) for each of the tested samples. Quantitative porosity analysis was also performed, the closed and open porosity fractions were determined. Performed calculations, both of the solid phase fraction and the porosity, were based on the numerical 3D analysis of chosen volumes in the reconstructed tomographic views of samples, allowed by the CTAn software. 

## 3. Results and Discussion

The properties of the foundry binders are presented in Table 3. 

The results for each type of binder vary significantly. The traditional ZKE binder has a highly acidic pH, so as the Remasol Plus binder. According to the data from Table 1 and Table 3, it can be noticed that the acidic binders were used for the first two layers, whilst alkaline binders were used for the structural layers. The ZKE binders have the smallest fraction of the solid phase, i.e., SiO_2_ nanoparticles, as well as the smallest relative viscosity, defined by the time of mass outflow from the Zahn cup No. 4.

The observations of solid-phase morphology suggest that the quartz meal used to prepare the spherical samples has irregularly shaped particles with sharp edges. The same is true for quartz sand with particle sizes ranging from 0.1 to 0.3 mm. The use of a solid phase with this type of morphology ensures better form compactness in the modelling layer (Layer 1) and sealing layer (Layer 2). The sand with the largest particle diameter, from 0.5–1.0 mm, has irregular but rounded edges which ensure expected open porosity and gas permeability.

**Figure 1:**
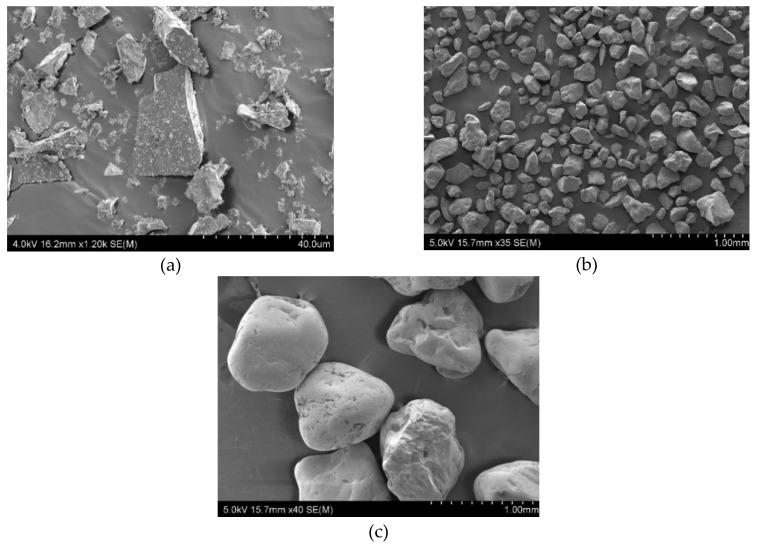
Morphology of powder particles used in the sample molds: (**a**) quartz meal; (**b**) silica sand 0.1–0.3 mm; and (**c**) silica sand 0.5–1.0 mm.

Figure 1 shows images of the granular material taken using a scanning electron microscope, SU70.

**Figure 2:**
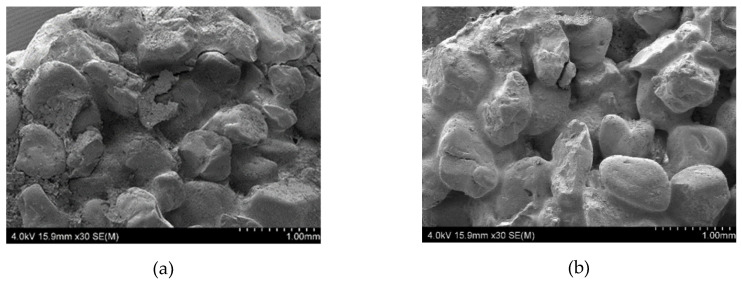
The scanning electron microscopy (SEM) morphology of the ceramic mold samples: (**a**) Sample Q1 and (**b**) Sample Q2.

Figure 2 shows the morphology of the raw mold samples at their fractures. The SEM photos show that the binder is surrounded by the particles of meal and sprinkles of quartz sand. For the Q1 sample, discontinuities, defects, and cracks are visible at the grain-binder/quartz meal boundary. This is due to non-ideal properties of binders resulting in a decrease of the adhesion and wettability of the quartz particles with the ZKE binder. This results in the defects induced by a lower concentration of nano SiO_2_ in the ZKE binder and its high acidity.

The addition of water-based binders containing colloidal silica ensures a tight and even coverage of the coarse grains by the binder/quartz meal composition. What is more, there are visible bridges connecting the quartz sand particles. 

**Figure 3:**
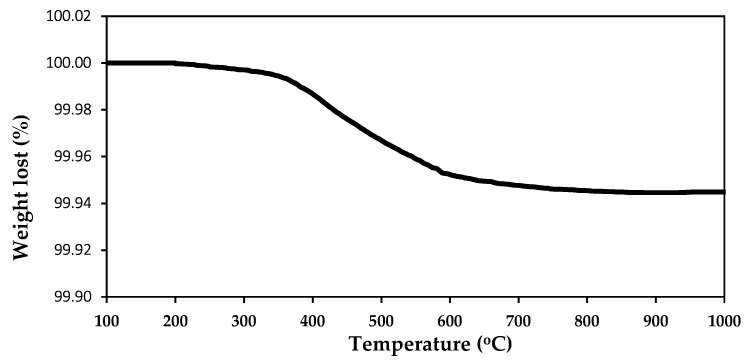
TGA curve of silica powder.

The TGA results of quartz meal are shown on the Figure 3.

The analyzed quartz sand has a high thermal stability. Its mass was constant up to 380 °C. In the temperature range between 380 and 600 °C a minor mass decrease was observed, related to the dihydroxylation of SiO_2_. Very low mass loss of the quartz meal is the evidence of its high purity and lack of organic impurities. TGA results of silica sprinkle were analogical. 

The powders exhibited high clarity, as confirmed by the chemical composition (XRF, Karlsruhe, Germany) results presented in Table 4. The total contaminants present in silica powder did not exceed 0.22 wt.% and included aluminum in the form of Al_2_O_3_ (0.14 wt.%), iron in the form of Fe_2_O_3_ (0.07 wt.%).

**Figure 4:**
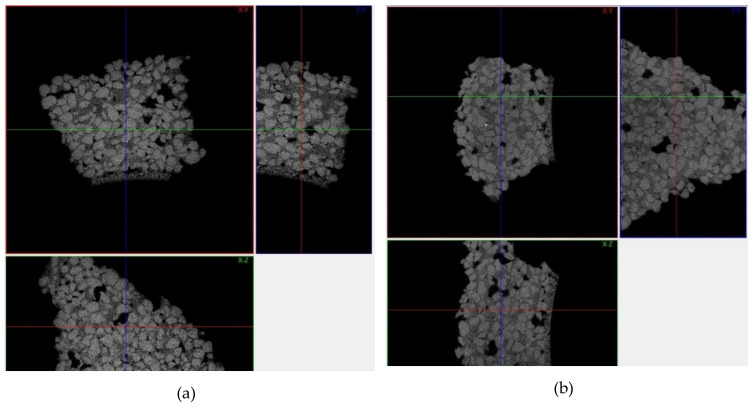
Ceramic mold sample tomograms with the three cross-sections shown in the XY, YZ, and XZ axes for (**a**) Sample Q1 and (**b**) Sample Q2.

**Figure 5:**
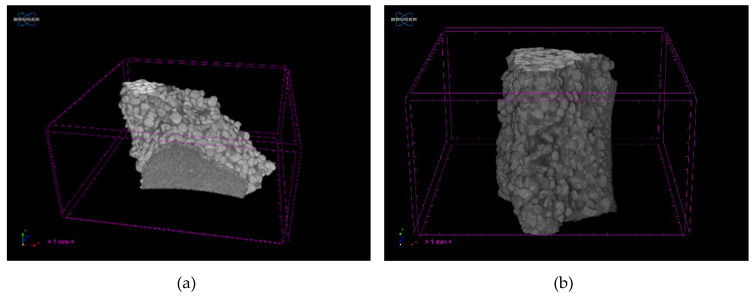
Three-dimensional reconstructions of the raw samples (**a**) Q1 and (**b**) Q2.

Figure 4 shows the tomograms of the raw Q1 and Q2 samples, while Figure 5 shows their three-dimensional reconstructions. Obtained tomographic results, both the tomograms and reconstructions, show that the raw samples have similar morphology and defects.

Table 5 contains the porosity measurements and solid-phase percentage of the raw samples.

The tomographic calculations indicate that samples Q1 and Q2 have different values of open porosity and solid-phase particle share, both values are over 2% higher for Q2. Even such minor differences affect the parameters of the raw molds, as well as molds after heat treatment. Gas permeability of the shell molds was particularly dependent on the results of the open porosity measurements. As expected, higher rate of porosity resulted in a greater gas permeability of the Q2 sample, as compared to the Q1 sample.

The samples obtained using an “immersion-sprinkling” method were subjected to thermal treatment at 950 °C/h in a chamber furnace, with the furnace heated at a rate of 5 °C/min. After reaching the annealing temperature, the samples were cooled in the furnace to avoid cracks.

**Figure 6:**
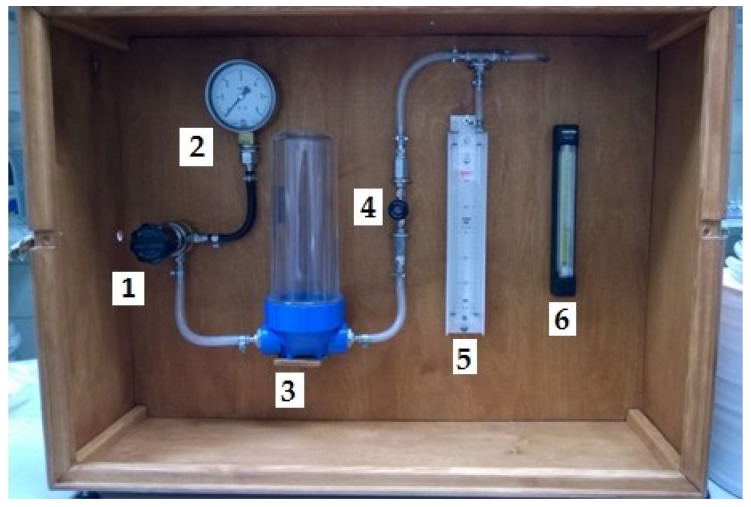
Gas permeability measuring station for the ceramic molds, where: (1) diaphragm valve, (2) circular manometer, (3) dryer, (4) needle valve, (5) liquid manometer, and (6) flowmeter.

Gas permeability measurements were performed with a use of a homebuilt testing device. An outline of the device is shown in the Figure 6. 

The system is powered by compressed air supplied from a cylinder on the diaphragm valve side. The sample is located at the end of the system behind the flow meter. The diaphragm valve (1) is connected to the pressure gauge (2) and the air dryer (3). The air dryer outlet is connected to a needle valve (4), which precisely regulates the pressure of air reaching the liquid manometer (5) and flow meter (6). The flow meter outlet is connected to the sample. The airflow and pressure readout from the liquid manometer are recorded during the measurements.

Darcy’s law was utilized to calculate the permeability (*K*) [19,20,21,22,23,24]:

(1) $$K=\frac{MQ\prime D}{PA}$$

*K*—gas permeability (cm^2^);
*M*—dynamic air viscosity at a given temperature (Figure 3) ((kg/m·s) 10^−5^);
*D*—wall thickness of the mould (mould diameter—ping-pong ball diameter) (mm);
*A*—mould surface area (mm^2^);
*P*—air pressure (mm H_2_O);
*Q’*—airflow value at measurement temperature (cm^3^/min).

The dynamic air viscosity (M), in the range of temperatures from 20 to 1300 °C, is directly proportional (linear correlation) to the temperature and the measurement is restricted to this range of temperatures. The dynamic viscosity value for a particular temperature can be directly readout from the device.

The airflow value is dependent on the temperature. A laboratory chamber furnace is used for measurements in the media at different temperatures. Therefore, air as a working medium also has varying temperatures. Considering this, a correction should be made to the airflow values at the measurement temperature as described in Equation (2):

(2) $$Q\prime =\frac{QT\prime }{T}$$

where:

*Q’*—airflow value at measuring temperature (cm^3^/min);
*Q*—airflow value at room temperature (cm^3^/min);
*T’*—measurement temperature value (°C);
*T*—room temperature value (°C).

**Figure 7:**
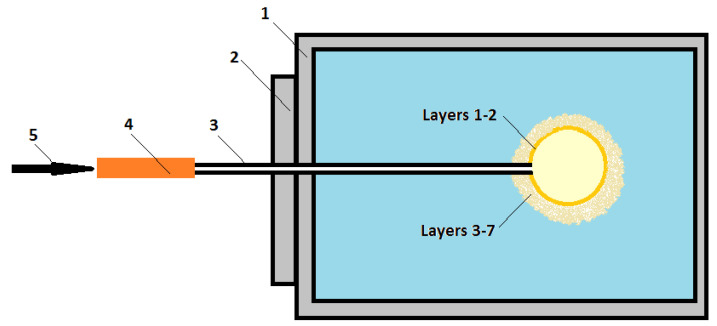
Arrangement of the sample and air providing system in the furnace, where: (1) furnace chamber, (2) furnace door, (3) sample, (4) rubber pipe, and (5) air inlet.

Figure 7 presents an arrangement of the sample and air providing system in the furnace.

Samples for the gas permeability measurements were installed in the high temperature furnace. Sample (3) consists of a quartz pipe and a spherical, porous ceramic part. Spherical part is placed in the furnace chamber and the quartz pipe is located in the furnace door. The air is supplied to the sample through the measuring station from a cylinder linked with a rubber pipe, as shown in the Figure 6. The air flow rate was 30 cm^3^/min. Gas permeability measurements at elevated temperatures are possible in this device. Therefore it allows obtaining results as close as possible to the working conditions of the ceramic mold. The gas permeability tests on samples previously subjected to the furnace process were conducted at temperatures ranging from 25 to 950 °C.

Table 6 shows the gas permeability results of the tested mold samples. The results of the shell mold samples were calculated as a mean value of five measurements. The gas permeability of ceramic mold samples increases with the increasing temperature. The increase of the gas permeability is minor in the temperatures between 25 and 400 °C. Deviations become noticeable at higher temperatures. Obtained results are related with the polymorphic transitions of quartz. α quartz → β-quartz transition taking place in 573 °C effects in the expansion of material, while β-quartz → β-tridymite transition in approximately 870 °C results in the 14.4% increase of its volume [25,26,27]. Such transformations impose the increase of pore size and the rate of gas permeability.

Sample Q2 made with Remasol Plus and Remasol Premium water-based binders shows a higher gas and air permeability over the entire range of temperatures. It can be concluded, that apart from the particle size and shape, gas permeability is also influenced by the type of binder. Replacing the alcohol and water system (ZKE–LUDOX PX 30) with solely water-based binder results in the formation of a porous material with higher gas permeability over a range of temperatures from 25 to 950 °C. The gas permeability values obtained within the examined range make the Q2 composition interesting and promising for practical purposes. ZKE is an alcoholic binder that can deteriorate the health of the person completing the casting molds. The use of Remasol water-based binders provides a solution that will not affect the health of employees and offers a chance to completely replace molding systems based on ZKE [28,29,30,31].

**Figure 8:**
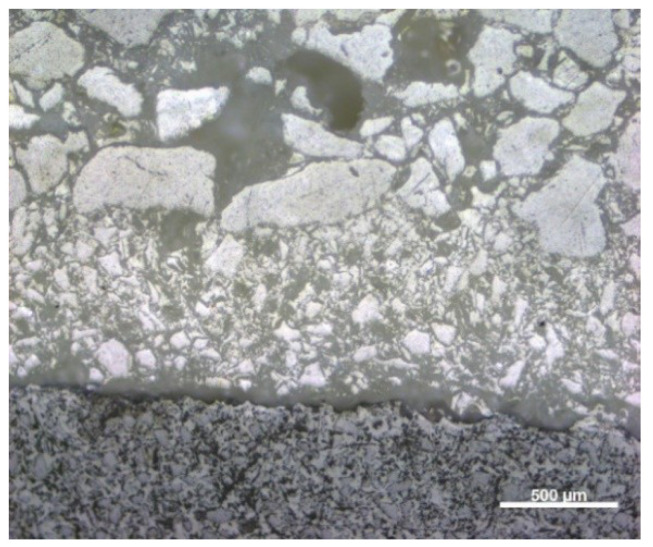
Microstructure of fracture: sample Q1, heated at 950 °C (light microscopy).

**Figure 9:**
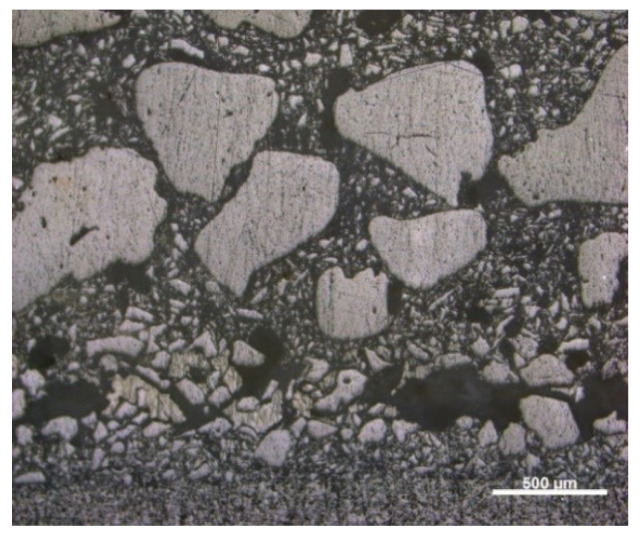
Microstructure of fracture: sample Q2, heated at 950 °C (light microscopy).

Figure 8 and Figure 9 show the microstructures of fractures of samples Q1 and Q2, which show the applied layers of the tested materials.

The first and second layer are most noticeable in both samples. However, distinguishing between construction layers after the thermal treatment in 950 °C is barely possible due to the high contribution of the SiO_2_ in the mold’s and cast’s solid phase. The results of microtomography did not expose particular layers as well

**Figure 10:**
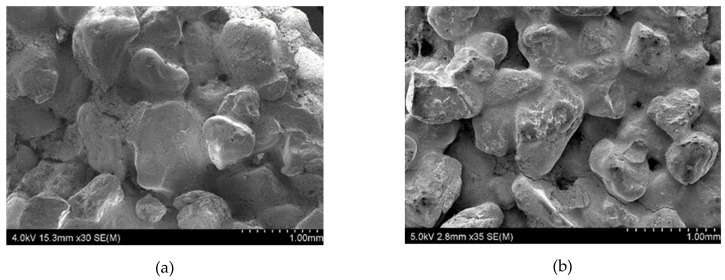
SEM morphology of the ceramic mold samples heated to 950 °C for: (**a**) Sample Q1 and (**b**) Sample Q2.

**Figure 11:**
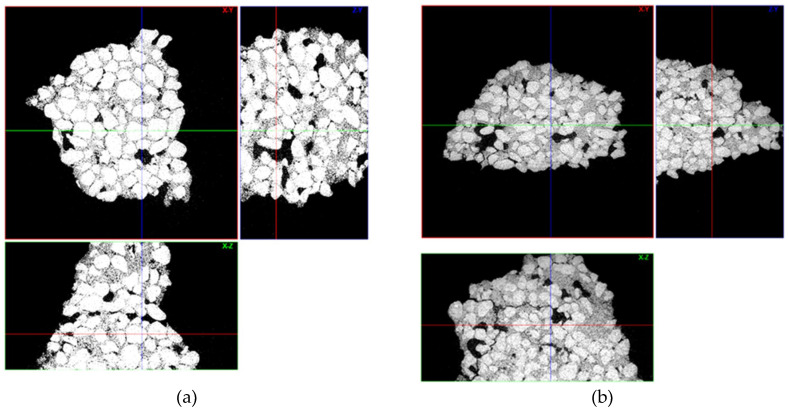
Ceramic mold sample tomograms heated at 950 °C, showing three cross-sections in the XY, YZ, and XZ planes for: (**a**) Sample Q1 and (**b**) Sample Q2.

**Figure 12:**
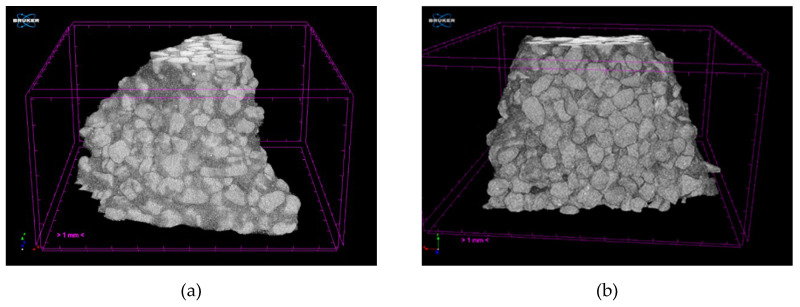
Three-dimensional reconstructions of: (**a**) Sample Q1 and (**b**) Sample Q2 post heating at 950 °C.

The type of binders used and their interaction with powder particles and the differences in the porosity of the tested samples are the main reasons of variation in the gas permeability values. The morphology of the samples Q1 and Q2 at their fracture sites is shown in the Figure 10. The tomograms of samples Q1 and Q2 after heating to 950 °C are shown in the Figure 11 and their three-dimensional reconstructions are given in the Figure 12. Sample Q1, after being heated at 950 °C, has a more compact structure than Sample Q2, in which single macroscopic defects are visible. Binder ZKE produces a uniform coating for grains with ceramic material and produces less defective samples (Figure 11a), in comparison with the samples obtained using Remasol binders (Figure 11b). Cooling process and the shrinkage hindering effect of the binders can also have an impact on the results presented in the Table 6.

These results are confirmed by the tomographic scans (Figure 10 and Figure 11). The Q2 sample contains cracks running along the boundaries of the quartz sprinkling particles. Pores in both materials are stochastic and comparable (Figure 10). The 3D reconstruction of the analyzed Q2 sample shows more open pores (Figure 11b).

The results of porosity and solid-phase share calculations of the samples heated at 950 °C are presented in the Table 7.

The results of the calculations show that the annealing temperature and the use of different binders influence the porosity of the multilayer molds. Open porosity of Sample Q2 is approximately twice as high, which results in a higher gas permeability, as compared to Sample Q1. The ceramic molds with Q2 composition can be used in industrial practice in the precision casting processes. Although, these types of molds need to be further investigated to determine their casting ability with liquid metal and under industrial conditions.

## 4. Conclusions

The properties of two types of seven-layer mold samples made with the use of the immersion- sprinkling method were compared. Sample Q1 was obtained using a combined alcohol system based on ZKE (layers 1, 2, 5 and 7) and a LUDOX PX 30 water binder (layers 3, 4, and 6). Sample Q2 was made entirely using a water system, only with Remasol binders. The type of binder affects the properties of the quartz sand casting mold samples. Sample Q2 has a higher open porosity in the raw state as well as after heating at 950 °C and what is more, after baking the open porosity increases from 7.51% to 12.23%. This is 5.62% higher than for sample Q1. 

The higher open porosity of the Q2 mold did not double the gas permeability, as compared to Q1. Gas permeability of the spherical mold Q2 was 9.89 × 10^−14^ m^2^, whereas in the mold Q1 it had a similar value of 8.42 × 10^−14^ m^2^. Comparing both systems it can be assumed that the Q2 has better and more promising properties than Q1. Industrial testing coupled with an evaluation of the breakout and casting is still required before industrial application can be achieved.

The presented research is of the application character. The potential of employing the Q2 water system in the industrial conditions has been proved. Further research on controlling the cooling process of the molds, increasing properties such as heat resistance, creep resistance or bending strength, and different metals such as cast iron or nickel superalloys is planned.

## Figures and Tables

**Table 1:** Raw material compositions of the produced samples.

Sample	Layer	Molding Compound	Sprinkling
Q1	1	ZKE + silica powder	Silica sand 0.1–0.3 mm
	2	ZKE + silica powder	Silica sand 0.1–0.3 mm
	3	LUDOX PX 30 + silica powder	Silica sand 0.5–1.0 mm
	4–7	Alternatively Ludox PX 30 + silica powder and ZKE + silica powder	Silica sand 0.5–1.0 mm
Q2	1	Remasol Plus + silica powder	Silica sand 0.1–0.3 mm
	2	Remasol Plus + silica powder	Silica sand 0.1–0.3 mm
	3–7	Remasol Premium + silica powder	Silica sand 0.5–1.0 mm

**Table 2:** Scanning parameters for the ceramic casting mould samples.

Lamp Voltage (kV)	Current (µA)	Filter	Measurement Step (°)	Exposure Time (ms)	Pixel Size (µm)
100	100	A–Cu	0.20	500	10.99

**Table 3:** Properties of polymeric binders.

Molding Binder	pH	C (%)	Outflow Time (s)
ZKE	1.51	24.63	5.92
Ludox PX 30	9.83	33.52	6.45
Remasol Plus	5.93	30.79	6.88
Remasol Premium	10.78	30.66	6.12

**Table 4:** Chemical composition of silica powder.

Element	Concentration (wt.%)
Si	99.78
Al	0.14
Fe	0.07
Ti	<15 ppm
Ca	<15 ppm
K	~10 ppm
Na	~10 ppm

**Table 5:** Porosity and percentage of particles in the raw samples Q1 and Q2.

Sample	Open Porosity (%)	Closed Porosity (%)	Particle Share (%)
Q1	5.42	1.73	43.33
Q2	7.51	1.74	46.12

**Table 6:** Gas permeability of the ceramic samples, Q1 and Q2.

Temperature (°C)	Gas Permeability K (m^2^)	
	Q1	Q2
25	4.99 × 10^−14^ ± 0.11	5.26 × 10^−14^ ± 0.07
400	5.73 × 10^−14^ ± 0.13	5.98 × 10^−14^ ± 0.08
650	6.35 × 10^−14^ ± 0.17	6.87 × 10^−14^ ± 0.12
850	7.11 × 10^−14^ ± 0.17	8.14 × 10^−14^ ± 0.16
950	8.42 × 10^−14^ ± 0.22	9.89 × 10^−14^ ± 0.19

**Table 7:** Porosity and percentage of particles in Samples Q1 and Q2 after heating at 950 °C.

Sample	Open Porosity (%)	Closed Porosity (%)	Particle Share (%)
Q1	6.61	1.82	50.93
Q2	12.23	1.31	48.87

## References

[1] Hong J., Ma D., Wang J., Wang F., Sun B., Dong A., Li F., Bührig-Polaczek A. (2016). Freckle Defect Formation near the Casting Interfaces of Directionally Solidified Superalloys. Materials.

[2] Zhang H., Xu Q. (2017). Simulation and Experimental Studies on Grain Selection and Structure Design of the Spiral Selector for Casting Single Crystal Ni-Based Superalloy. Materials.

[3] Szeliga D., Ziaja W., Motyka M., Kubiak K., Sieniawski J. (2019). Application of Inner Radiation Baffles in the Bridgman Process for Flattening the Temperature Profile and Controlling the Columnar Grain Structure of Directionally Solidified Ni-Based Superalloys. Materials.

[4] Wiśniewski P. (2020). Evaluating silicon carbide-based slurries and molds for the manufacture of aircraft turbine components. Crystals.

[5] Huang P., Lu G., Yan Q., Mao P. (2019). Effect of Ceramic and Nylon Fiber Content on Composite Silica Sol Slurry Properties and Bending Strength of Investment Casting Shell. Materials.

[6] Nadolski M., Konopka Z., Lagiewka M., Zyska A. (2008). Examining of slurries and production of moulds by spraying method in lost wax technology. Arch. Foundry Eng..

[7] Małek M., Wiśniewski P., Matysiak H., Kurzydłowski K.J. (2015). Technological properties of ceramic slurries based on aluminium III oxide for ceramic shell moulds fabrication. Mechanik.

[8] Ferenc J., Matysiak H., Kurzydłowski K.J. (2010). Organic Viscosity Modifiers for Controlling Rheology of Ceramic Slurries Used in the Investment Casting. Adv. Sci. Technol..

[9] Pattnaik S., Karunakar D.B., Jha P.K. (2012). Developments in investment casting process—A review. J. Mater. Process. Technol..

[10] Wiśniewski P., Sitek R., Koralnik M.K., Spychalski W.L., Moszczyńska D., Mizera J. (2017). Badania procesu studzenia próbek ceramicznych form odlewniczych z zastosowaniem kamery termowizyjnej. Mater. Ceram..

[11] Małek M., Wiśniewski P., Matysiak H., Zagórska M., Kurzydłowski K.J. (2014). Technological properties of SiC-based ceramic slurries for manufacturing investment casting shell moulds. Arch. Met. Mater..

[12] Tomasik J., Haratym R., Biernacki R. (2009). Investment casting or powder metallurgy—The ecological aspect. Arch. Foundry Eng..

[13] Małek M., Wiśniewski P., Szymańska J., Mizera J., Kurzydłowski K.J. (2016). Technological Properties of Ceramic Slurries Based on Silicon Carbide with Poly (vinyl alcohol) Addition for Shell Moulds Fabrication in Precision Casting Process. Acta Phys. Pol. A.

[14] Reed R.C. (2006). The Superalloys, Fundamentals and Applications.

[15] Jones S., Yuan C. (2003). Advances in shell moulding for investment casting. J. Mater. Process. Technol..

[16] Haratym R., Biernacki R., Myszka D. (2008). Ekologiczne Wytwarzanie Dokładnych Odlewów W Formach Ceramicznych.

[17] Haratym R. (1997). Procesy Odlewania Precyzyjnego W Formy Odlewnicze.

[18] Wiśniewski P., Małek M., Mizera J., Kurzydłowski K.J. (2017). Effect of adding water-based binders on the technological properties of ceramic slurries based on silicon carbide. Mater. Technol..

[19] Amira S., Dube D., Treamblay R. (2011). Method to determine hot permeability and strength of ceramic shell moulds. J. Mater. Process. Technol..

[20] Koralnik M.K. (2014). Badanie Porowatości I Gazo-Przepuszczalności Form Ceramicznych Na Bazie Proszków Molochite Do Odlewania Precyzyjnego Stopów Niklu. Bachelor’s Thesis.

[21] Koralnik M.K. (2015). Research and Modifications Ceramic Molds to Receive Large-Size Aircraft Engine Parts. Master’s Thesis.

[22] Kumar S., Karunakar D.B. (2020). Characterization and Properties of Ceramic Shells in Investment Casting Process. Int. J. Met..

[23] Sanjay K., Karunakar D.B. (2019). Enhancing the permeability and properties of ceramic shell in investment casting process using ABS powder and needle coke. Int. J. Met..

[24] Małek M. (2016). Opracowanie Ekologicznej Technologii Wytwarzania Form Ceramicznych Do Odlewania Precyzyjnego Łopatek Turbin Silników Lotniczych Z Nadstopów Niklu. Ph.D. Thesis.

[25] Grimm H., Dorner B. (1975). On the mechanism of the α-β phase transformation of quartz. J. Phys. Chem. Solids.

[26] Ghiorso M.S., Carmichael I.S.E., Moret L.K. (1979). Inverted high-temperature quartz. Contrib. Miner. Pet..

[27] Razafinjato R.N., Beaucour A.-L., Hebert R.L., Ledeser B., Bodet R., Noumowe A. (2016). High temperature behaviour of a wide petrographic range of siliceous and calcareous aggregates for concretes. Constr. Build. Mater..

[28] Wang F., Li F., He B., Wang D., Sun B. (2013). Gel-casting of fused silica based core packing for investment casting using silica sol as a binder. J. Eur. Ceram. Soc..

[29] Omar M.F.M., Sharif S., Ibrahim M., Fadzil A.S.A., Mohd A.A. (2014). Diferential ceramic shell thickness evaluation for direct rapid investment casting. Appl. Mech. Mater..

[30] Ospennikova O.G., Pikulina L.V., Antipin L.M. (2010). Application of a Hydrolyzed Ethyl Silicate in Investment Casting. Inorg. Mater..

[31] Ismael M.R., Anjos D.R., Salomao R., Pandolfelli V.C. (2006). Colloidal silica as a nostructured binder for refractory castables. Refract. Appl. News.

